# Secular trends of low birth weight, preterm birth, and small for gestational age in Shanghai from 2004 to 2020: an age-period-cohort analysis

**DOI:** 10.1186/s12884-023-05799-9

**Published:** 2023-07-26

**Authors:** Rongfei Zhou, Huiting Yu, Naisi Qian, Shan Jin, Renzhi Cai, Lei Chen, Chunfang Wang, Fan Wu

**Affiliations:** 1 School of Public Health, Fudan University, Shanghai, 200032 China; 2 Vital Statistical Department, Shanghai Municipal Center for Disease Control and Prevention, Institute of Health Information, Shanghai, 200336 China

**Keywords:** Epidemiology, Preterm birth, Small for gestational age, Age-period-cohort analysis

## Abstract

**Background:**

Although highly heterogeneous among countries, the incidence rates of low birth weight (LBW), preterm birth (PTB), and small for gestational age (SGA) have been increasing globally over the past two decades. To better understand the cause of these secular trends, this study aimed to investigate the effects of age, period, and birth cohort on LBW, PTB, and SGA rates in Shanghai.

**Methods:**

Data from 2,958,695 singleton live births at 24–41 gestational weeks between 2004 and 2020 were obtained for this study. Age-period-cohort models based on Poisson regression were used to evaluate the independent effects of maternal age, delivery period, and maternal birth cohort on the trends in LBW, PTB, and SGA.

**Results:**

The overall prevalence rates of LBW, PTB, and SGA were 2.9%, 4.7%, and 9.3%, respectively, and significant changes were observed (average annual change: + 10.7‰, + 9.1‰, -11.9‰) from 2004 to 2020. Cohort effect increased steadily, from 1960 (risk ratio [RR] = 0.71, 95% confidence interval [CI]: 0.65–0.78) to 1993 (RR = 0.97, 95% CI: 0.94–1.01) for LBW and from 1960 (RR = 0.69, 95% CI: 0.64–0.75) to 2004 (RR = 1.02, 95% CI: 0.94–1.12) for PTB. A strong cohort effect was found with the highest risk of SGA (RR = 1.82, 95% CI: 1.72–1.93) in 1960 and the lowest risk (RR = 0.57, 95% CI: 0.54–0.61) in 2004, compared with the reference cohort of 1985. There was a “U-shaped” maternal age effect on LBW and PTB and a weak period effect on the three birth outcomes.

**Conclusions:**

Our findings suggested a significant independent effect of age, period, and birth cohort on the three birth outcomes. The increasing rates of LBW and PTB motivated us to focus on young and advanced pregnant women. Meanwhile, the prevalence of SGA decreased steadily, illustrating the need for further research on the mechanisms underlying these trends.

**Supplementary Information:**

The online version contains supplementary material available at 10.1186/s12884-023-05799-9.

## Background

Adverse birth outcomes (ABOs), including low birth weight (LBW), preterm birth (PTB), small for gestational age (SGA), and stillbirths and miscarriage, were the leading causes of neonatal mortality and morbidity in young children [1–3]. Numerous studies reported ABOs as a significant global public health problem over the past two decades [1–3]. It was estimated that 12 million PTB and 32 million SGA babies were born in sub-Saharan African and South Asian countries, accounting for most of global ABOs [2, 4]. PTB rates were reported to be approximately 5% in Europe and 18% in Africa [4]. Kaforau et al. also estimated the mean prevalence rates of LBW and PTB in 11 countries in the Pacific region to be 12% and 13%, respectively [5]. South Asia had the highest rate of SGA, where 40% were categorised as SGA [6]. In China, the estimated prevalence rates of LBW, PTB, and SGA were 7.2%, 6.1%, and 12.3%, respectively [2, 7, 8]. These findings demonstrated that ABOs mainly occur in low- and middle-income countries and that ABO incidence rates were highly heterogeneous worldwide [4, 6].

LBW, referring to a birth weight < 2500 g, was a valuable marker of immaturity at delivery [1]. PTB, mostly defined as birth before 37 completed weeks of gestation, commonly led to neonatal mortality and morbidity [9]. SGA means the birth weight falls below a gestational age and sex-specific cut-off point, which was commonly the lowest 10th centile or 2 standard deviations (SDs) below the average [10, 11]. Therefore, SGA can be considered a retrospective indicator of intrauterine growth restriction [3]. Infants born with ABOs were at an increased risk of respiratory distress syndrome, stunting, mental retardation, and early childhood mortality [6].

Since the Reform and Opening up in the 1990s, Chinese people have undergone a dramatic economic and nutritional transition [12]. Along with changes in sociodemographic and individual characteristics of pregnant women, such as shifting to an urban lifestyle (residence in urban areas, sedentary behaviour and increased mental pressure), older age of delivery (≥ 35 years) and a higher level of maternal education, the epidemiology of ABOs has also changed [13, 14]. These changes may contribute to the future burden of chronic diseases, given the potential risks of fetal growth restriction. Previous studies have established how maternal age has a “U-shaped” effect on ABOs [15, 16]. Although studies have examined secular trends in PTB and SGA in China, these analyses used either age or period as an additional factor [8, 14]. Age-period-cohort (APC) analysis was a classic model used to demonstrate trends in health outcomes because it can simultaneously examine the effect of maternal age (age effect, defined as variations caused by physiological changes and social status changes) [17], delivery year (period effect, representing a set of social events and environmental factors such as medical technology and public health policies before outcomes), and maternal birth year (cohort effect, reflecting individual experience and exposure factors during their lifetime) [18]. To better understand the cause of the secular trends, this study aimed to clarify the effects of maternal age, period of delivery, and maternal birth cohort on LBW, PTB, and SGA in Shanghai.

## Methods

### Study population

Birth data from the year 2004 to 2020 was collected from the birth registry system of the Shanghai Municipal Centre for Disease Control and Prevention (SCDC), which was established in 2003 and covers all hospitals with authorized delivery services in Shanghai. After excluding twin or multiple births (*n* = 81,120, 2.62%); those with missing sex, parity, or maternal education data (*n* = 754, 0.03%); gestational age < 24^+0^ weeks or > 41^+6^ weeks (*n* = 39,277, 1.27%); outliers with ≥ 3 SDs from the gestational age; and sex-specific mean birthweight (*n* = 18,253, 0.59%), a total of 2,958,695 singleton live births were included in the data analysis. The flow of the study population selection is shown in Supplementary Fig. 1 [see Additional file].

### Definition of LBW, PTB, and SGA

The main ABOs investigated in this study included LBW, PTB, and SGA. According to previous studies, LBW was defined as a birth weight of less than 2500 g [5]. PTB was defined as delivery before the 37 completed weeks of gestation (or 259 days) [9], and SGA was defined as a birth weight < 10th centile for gestational age, gender-specific reference [10]. The reference of birth weight percentiles created by Mikolajczyk was adopted in this study [19], which could be adapted to the local population conveniently, without losing the predictive ability of ABOs. After identifying the mean birth weight and SD at 40 weeks, we obtained the birth weight percentiles according to the assumption of normal distribution for gestational age between 24 and 41 weeks. We excluded births at very early or late gestational ages based on the birth weight percentiles used for SGA, as described above. The birth weight percentiles are shown in Supplementary Table 1 [see Additional file].

### Statistical analysis

Maternal and neonatal characteristics, including maternal age, educational attainment, gravidity, parity, birth weight, gestational age, and incidence rates of LBW, PTB, and SGA, were analysed over a 5-year period. The age-standardised rate (ASR) was calculated by direct standardisation of the entire study population.

We used APC models to evaluate the net effects of maternal age, delivery period, and maternal birth cohort on the trends in LBW, PTB, and SGA based on the Poisson log-linear regression model. To resolve collinearity among age, period, and cohort (C = P-A), the method proposed by Carstensen was used [20]. Because of the small proportion of younger and older pregnant women (aged < 15, 0.07‰ and > 44, 0.62‰), maternal age < 15 was recoded as 15, and maternal age > 44 was recoded as 44. For visualization of trends, study populations were then categorised into 5-year age groups (15–19, 20–24, 25–29, 30–34, 35–39, and 40–44) and 5-year calendar period groups (2004–2008, 2009–2013, 2014–2018, and 2019–2020) according to their maternal age and date of delivery, respectively, and the birth cohort was computed by subtracting maternal age from the period.

Five sub-models were derived from APC modelling, including age, age–drift, age–cohort, age–period, and APC models. Overall linear trends, interpreted as estimated average annual changes, were extracted from the ‘drift’ variable in age-drift models. The model goodness-of-fit was evaluated based on residual deviance statistics. We examined the significance of pairwise comparisons of the sub-models using χ^2^ tests. Stratified APC models based on parity were also performed. All statistical tests were two-sided and P-values < 0.05 were considered significant, using the APC-fit function in the Epi package in R (version 4.1.0) [21].

## Results

The maternal and neonatal characteristics of the study population according to the delivery period were presented in Table 1. We included 295,8695 singleton live births, of which 52.9% were males and 47.1% were females. Maternal age at childbearing increased significantly, with a mean age of 26.9 years in 2004, which increased to 30.1 years in 2020. The percentage of highly educated mothers increased over time, whereas the proportion of multiparous mothers increased from a quarter to more than a third. However, there was no clear trend of change in birth weight during this period, although a very small decrease was observed.

**Table 1:** Maternal and neonatal characteristics by period

Characteristics	Total	2004–2008	2009–2013	2014–2018	2019–2020
	*n* = 2,958,695	*n* = 708,808	*n* = 975,772	*n* = 980,609	*n* = 293,506
Maternal characteristics					
Age (years, mean $\pm \mathrm{SD}$)	28.0 $\pm$ 4.7	26.9 $\pm$ 4.6	27.3 $\pm$ 4.7	28.9 $\pm$ 4.6	30.1 $\pm$ 4.4
Education (n, %)					
secondary and below	1,477,447 (49.9)	490,530 (69.2)	530,592 (54.4)	373,030 (38.0)	83,295 (28.4)
tertiary	1,481,248 (50.1)	218,278 (30.8)	445,180 (45.6)	607,579 (62.0)	210,211 (71.6)
Gravidity (n, %)					
1	1,365,259 (46.1)	337,315 (47.6)	469,150 (48.1)	431,993 (44.1)	126,801 (43.2)
2	837,105 (28.3)	209,026 (29.5)	266,389 (27.3)	277,072 (28.3)	84,618 (28.8)
≥ 3	756,331 (25.6)	162,467 (22.9)	240,233 (24.6)	271,544 (27.7)	82,087 (28.0)
Parity (n, %)					
0	2,033,081 (68.7)	532,336 (75.1)	697,308 (71.5)	620,668 (63.3)	182,769 (62.3)
1	844,240 (28.5)	161,191 (22.7)	248,971 (25.5)	333,350 (34.0)	100,728 (34.3)
≥ 2	81,374 (2.8)	15,281 (2.2)	29,493 (3.0)	26,591 (2.7)	10,009 (3.4)
Neonatal characteristics					
Sex (n, %)					
Male	1,565,963 (52.9)	378,860 (53.5)	518,926 (53.2)	515,056 (52.5)	153,121 (52.2)
Female	1,392,732 (47.1)	329,948 (46.5)	456,846 (46.8)	465,553 (47.5)	140,385 (47.8)
Birth weight (g, mean $\pm \mathrm{SD}$)	3,334.8 $\pm$ 442.8	3,340.6 $\pm$ 443.4	3,338.0 $\pm$ 441.0	3,333.1 $\pm$ 442.4	3,316.0 $\pm$ 447.7
Gestational age (days, mean $\pm \mathrm{SD}$)	273.3 $\pm$ 10.3	273.0 $\pm$ 10.4	272.4 $\pm$ 10.2	274.2 $\pm$ 10.1	273.7 $\pm$ 10.3
LBW (n, %)	84,899 (2.9)	19,372 (2.7)	26,822 (2.7)	28,888 (2.9)	9817 (3.3)
PTB (n, %)	138,961 (4.7)	31,014 (4.4)	43,814 (4.5)	48,293 (4.9)	15,840 (5.4)
SGA (n, %)	274,746 (9.3)	75,968 (10.7)	93,956 (9.6)	80,766 (8.2)	24,056 (8.2)

*Abbreviations*: *LBW* low birth weight, *PTB* preterm birth, *SGA* small for gestational age. LBW was defined as birth weight < 2500 g, PTB was defined as gestational age < 37 weeks, SGA was defined as birth weight below 10th centile for specific gestational age and sex

**Fig. 1:**
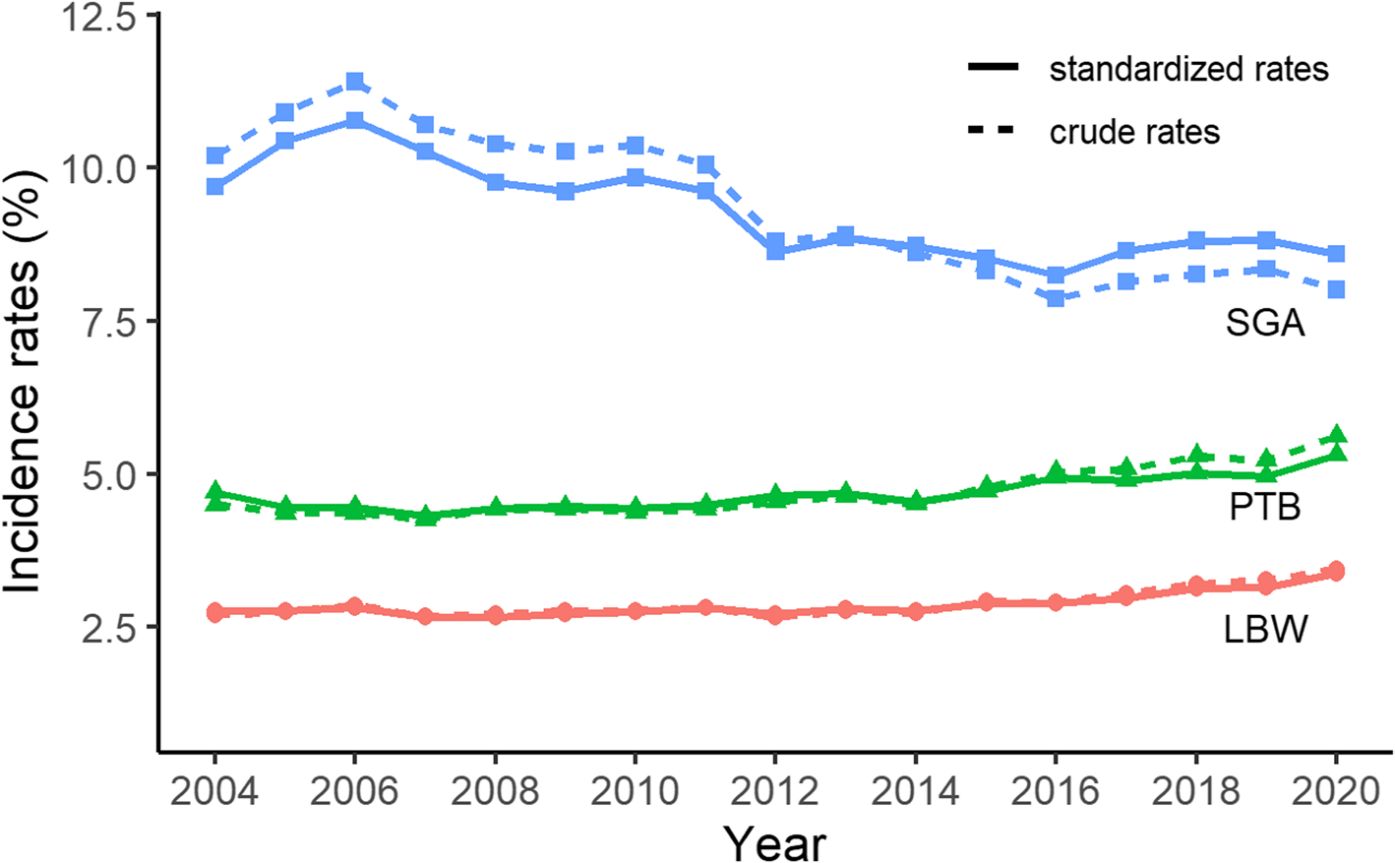
Crude rates and age-standardised rates of LBW, PTB, and SGA in Shanghai, 2004–2020

In the years from 2004 to 2020, the overall prevalence rates of LBW, PTB, and SGA were 2.9%, 4.7%, and 9.3%, respectively,. After standardisation, significant changes were observed in the trends of LBW, PTB, and SGA. The ASR for LBW increased from 2.8% in 2004 to 3.4% in 2020 with an average annual increase of 10.7‰ (95% confidence interval [CI], 9.2‰-12.2‰) and the ASR for PTB increased from 4.7% to 5.3% (annual increase: 9.1‰, 95% CI, 7.9‰-10.2‰), while the ASR for SGA declined from 9.7% to 8.6% (annual decrease: 11.9‰, 95% CI, 11.0‰-12.7‰). These trends are illustrated in Fig. 1.

### Specific trends for LBW, PTB, and SGA

**Fig. 2:**
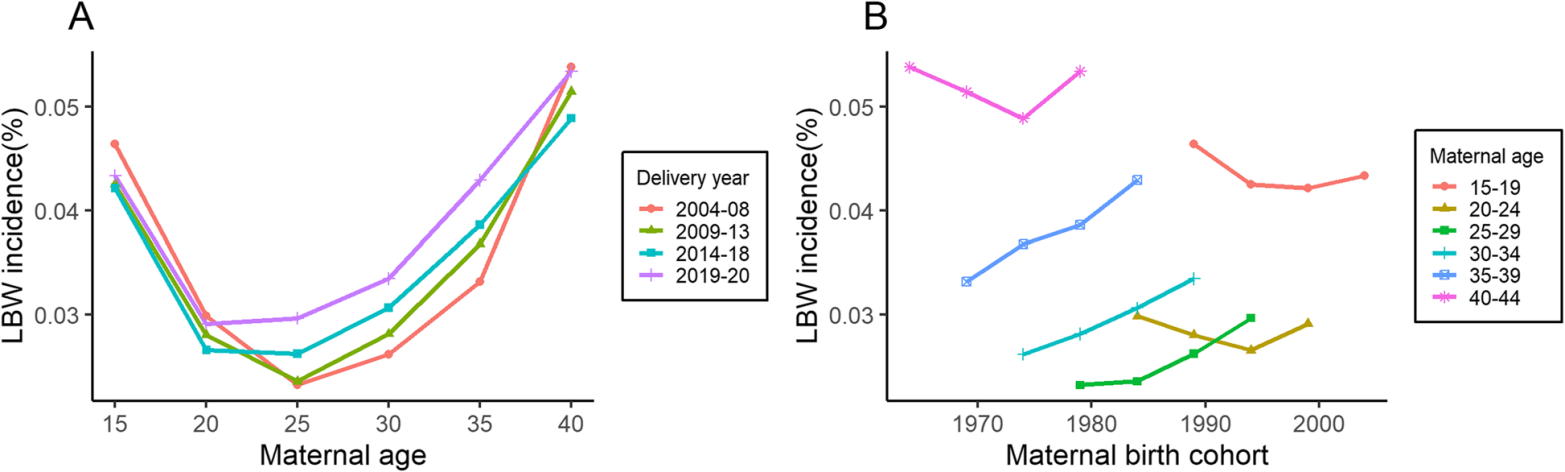
Specific incidence rates by (**A**) age and (**B**) cohort for LBW

To examine how the incidence rates of LBW differed by age and cohort, specific rates were plotted in Fig. 2. Age-specific incidence rates initially fell before age 25 years, then rose, resembling a “U-shaped” curve. Cohort-specific rates declined with the birth cohort, increased thereafter in the 15–24 years and 40–44 years age groups, and showed a steady upward trend in the 25–39 years age group.

**Fig. 3:**
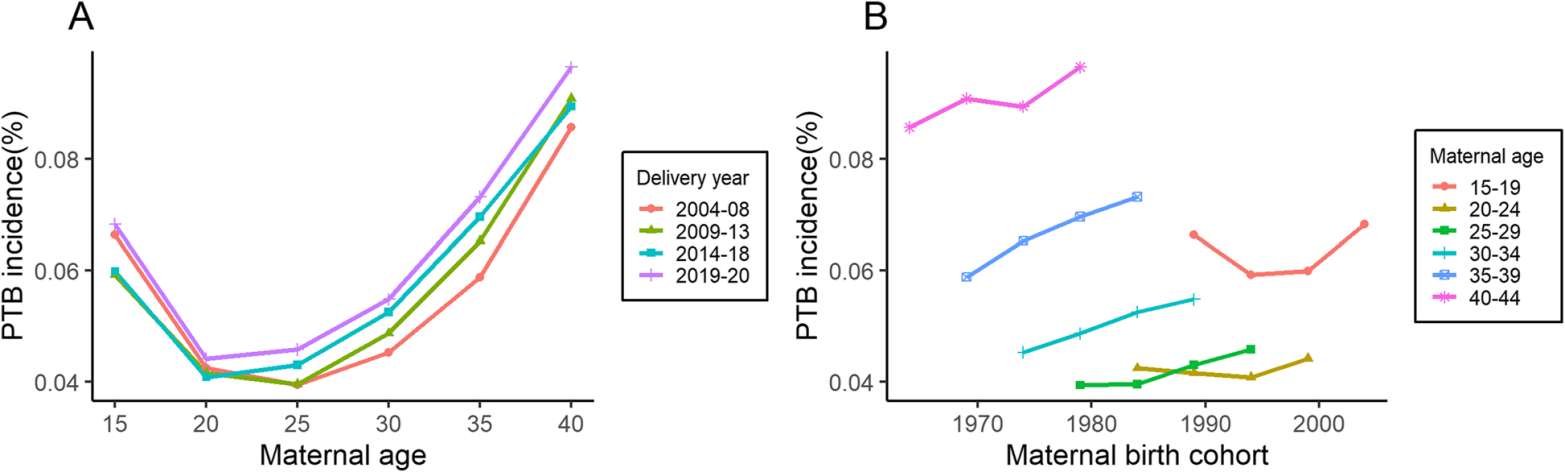
Specific incidence rates by (**A**) age and (**B**) cohort for PTB

Figure 3 showed the trends of PTB in different age, period, and cohort groups. Age-specific incidence rates displayed the same “U-shaped” variation in PTB, whereas cohort-specific rates increased steadily in all age groups, except the 15–19 years age group.

**Fig. 4:**
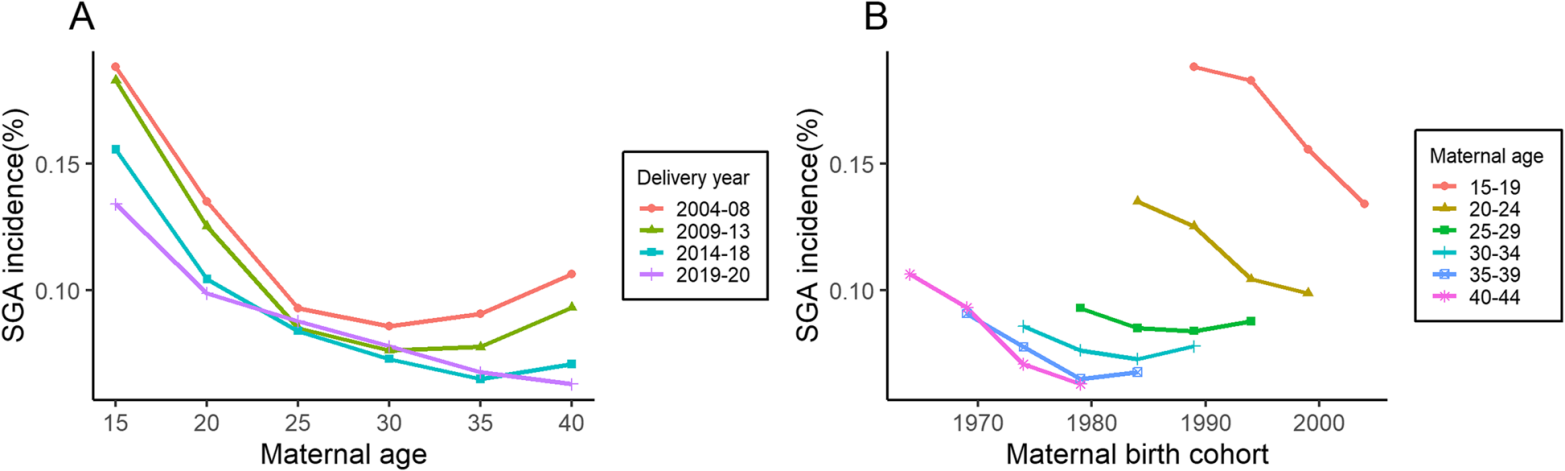
Specific incidence rates by (**A**) age and (**B**) cohort for SGA

The incidence rates of SGA according to age, period, and cohort were shown in Fig. 4. Overall, age-specific rates initially exhibited a decrease from age 15 to 35 years and then rose slightly after the age of 35 years. Rates among women aged 25–34 years remained stable over the entire period. However, for women in the younger or older age groups, the later the maternal birth cohort, the lower the incidence rate of SGA.

### APC effects for LBW, PTB, and SGA

**Fig. 5:**
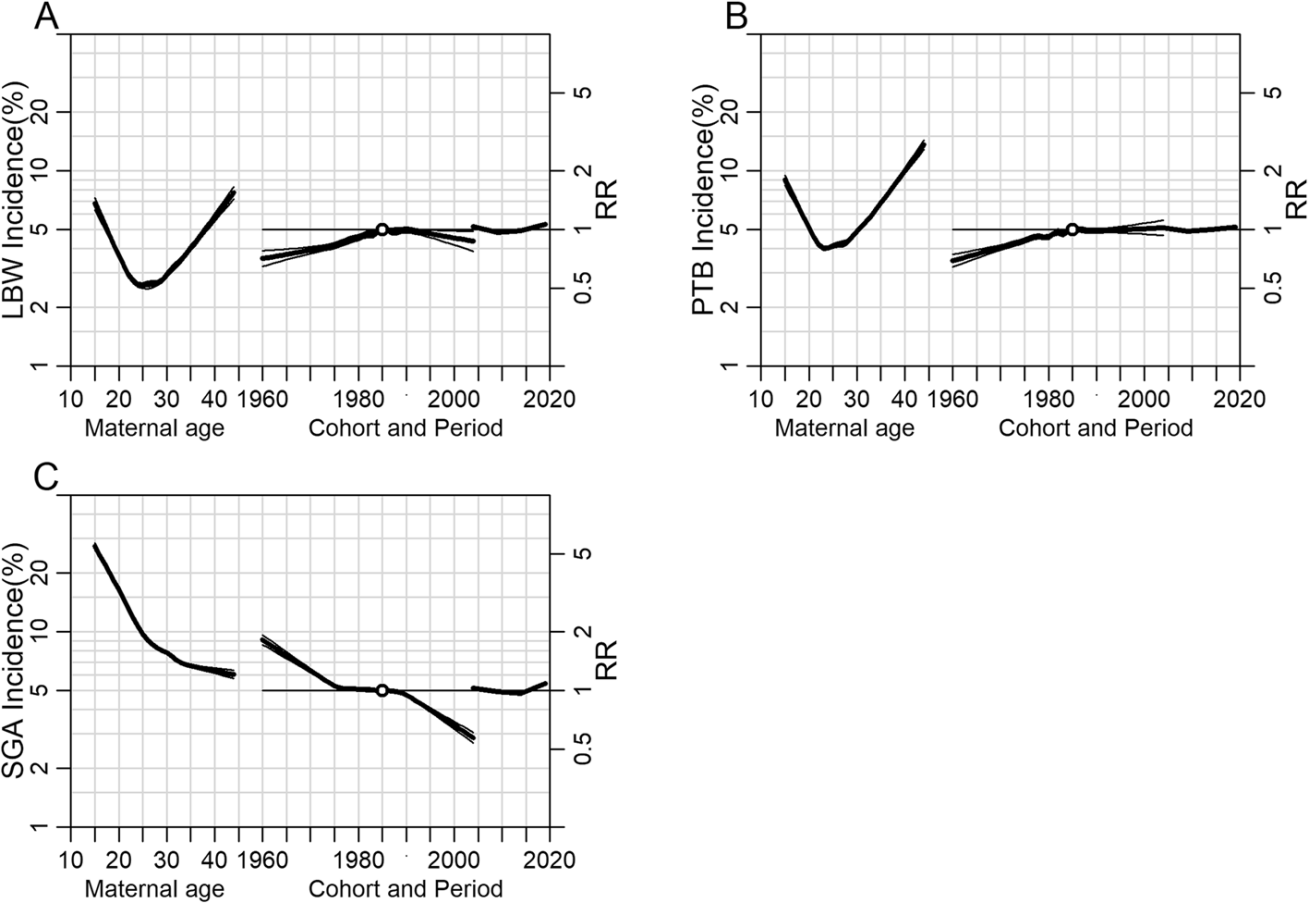
Age-period-cohort influences on trends in (**A**) LBW, (**B**) PTB, and (**C**) SGA. The left curve showed the fitted age-specific incidence at the reference cohort (1985), the middle curve was the risk ratios of cohorts relative to the reference cohort (1985), and the right curve was the risk ratios of period conditional on the estimated age and cohort effects

Figure 5 showed the estimated age, period, and birth cohort effects. Maternal age effect (the left curves) showed a changing trend in the incidence rate, while cohort and period effects (the middle and right curves) were illustrated by risk ratios (RR). Variation trend in the three effects indicates that age and birth cohort were the main risk factors for LBW, PTB, and SGA, whereas the period was relatively less impactful. The APC effects were similar among LBW and PTB births but different from those in SGA infants. The effect of age on the trends in LBW and PTB displayed a “U-shaped” curve, reaching its lowest value in the mid-20 s age group. The RR of birth cohort on LBW and PTB increased steadily before 1985 and then remained stable or declined slightly. When compared with the mothers born in 1985, those born in 1960 had the lowest RRs of 0.71 (95% CI, 0.65–0.78) for LBW, and 0.69 (95% CI, 0.64–0.75) for PTB. In contrast, a dramatic reduction in risk was observed in both age and cohort effects in SGA infants. The RRs of SGA decreased from 1.82 (95% CI, 1.72–1.93) to 0.57 (95% CI, 0.54–0.61) during the 40 years' cohort, compared with the reference cohort of those born in 1985. The RR of period effect on LBW, PTB, and SGA fluctuated around 1, without any obvious trend.

Stratified APC models performed separately by primiparas and multiparas showed the modification effects of parity on LBW, PTB, and SGA (see Additional file). The left curves of LBW incidence among different maternal age groups exhibited a similar “U-shaped” trend between primiparas and multiparas, where the incidence of LBW was reaching its nadir in the mid-20 s age group. When compared to the primiparous mothers born in 1985, the RR of cohort in LBW remarkably increased from 0.57 (95% CI, 0.51–0.65) in 1960 and then remained stable, whereas RR for multiparous mothers increased subtly from 0.88 (95% CI, 0.78–0.99) in the years from 1962 to 1985, but then fell to 0.62 (95% CI, 0.52–0.76) in 2003. The same modification effects of parity on APC models were also observed among PTB delivery. The incidence of PTB dropped to the lowest range around age 25, and the RRs began the rise from 1960 (RR, primiparas: 0.81, 95% CI, 0.73, 0.91; multiparas: 0.68, 95% CI, 0.61, 0.76) in both primiparous and multiparous mothers. When it came to SGA births, the incidence dropped dramatically with maternal age among both primiparous and multiparous mothers, and then increased slightly at advanced age (> 28) only in primiparous mothers. The RR of cohort increased from 0.72 (95% CI, 0.67–0.79) in 1960 before 1985 and then fell to 0.60 (95% CI, 0.56–0.64) in 2004 in primiparous mothers. Meanwhile, the RR of cohort maintained a consecutive decreasing trend from 3.35 (95% CI, 3.03–3.70) in 1960 to 0.46 (95% CI, 0.41–0.52) in 2003 among multiparous mothers. Overall, when compared with primiparous mothers aged over 25 years, the multiparous mothers had a lower incidence rate of SGA at the same age. These findings were presented in Supplementary Fig. 1, Supplementary Fig. 2 and Supplementary Fig. 3, respectively (see Additional file).

The age-period-cohort effects on the incidence rates of LBW, PTB, and SGA were evaluated using APC Poisson regression model. Comparisons of APC sub-models suggested that the full APC models were optimum, and incidence rates were significantly influenced by age and cohort effects when examining changes in residual deviance (Table 2). Age, period, and cohort effects, and their corresponding 95% Cis were described in Table 3.

**Table 2:** Comparisons of APC sub-models for LBW, PTB, and SGA

Model^a^	**Goodness of fit**				**Model comparison**				
	AIC	Residual Df	Residual Dev	Comparison	Change in Df	Change in Dev	Change in Dev/Df	*P*	Interpretation
LBW									
Age	1471.45	110	530.97						
Age-drift^b^	1272.41	109	329.93	2 versus 1	1	201.05	201.05	< 0.001	Trend (drift)
Age-Cohort	1241.33	101	282.85	3 versus 2	8	47.08	5.88	< 0.001	Nonlinear cohort effect
Age-Period-Cohort^c^	1160.83	99	198.35	4 versus 3	2	84.50	42.25	< 0.001	Period effect adjusted for cohort
Age-Period	1221.36	107	274.88	4 versus 5	8	76.53	9.57	< 0.001	Cohort effect adjusted for period
				5 versus 2	2	55.05	27.52	< 0.001	Nonlinear period effect
PTB									
Age	1476.57	110	476.48						
Age-drift^b^	1240.69	109	238.60	2 versus 1	1	237.88	237.88	< 0.001	Trend (drift)
Age-Cohort	1206.79	101	188.71	3 versus 2	8	49.90	6.24	< 0.001	Nonlinear cohort effect
Age-Period-Cohort^c^	1171.00	99	148.92	4 versus 3	2	39.79	19.89	< 0.001	Period effect adjusted for cohort
Age-Period	1222.87	107	216.79	4 versus 5	8	67.87	8.48	< 0.001	Cohort effect adjusted for period
				5 versus 2	2	21.82	10.91	< 0.001	Nonlinear period effect
SGA									
Age	2796.16	110	1736.37						
Age-drift^b^	2005.90	109	944.11	2 versus 1	1	792.26	792.26	< 0.001	Trend (drift)
Age-Cohort	1657.44	101	579.66	3 versus 2	8	364.46	45.56	< 0.001	Nonlinear cohort effect
Age-Period-Cohort^c^	1370.25	99	288.47	4 versus 3	2	291.19	145.59	< 0.001	Period effect adjusted for cohort
Age-Period	1785.60	107	719.82	4 versus 5	8	431.35	53.92	< 0.001	Cohort effect adjusted for period
				5 versus 2	2	224.30	112.15	< 0.001	Nonlinear period effect

*Abbreviations*: *APC* age-period-cohort, *LBW* low birth weight, *PTB* preterm birth, *SGA* small for gestational age, *AIC* Akaike information criterion, *Df* degree of freedom, *Dev* deviance

^a^ Models were ordered so that adjacent rows provided tests between models

^b^ The age-drift model was the intersection of the age–period and the age–cohort models

^c^ The best-fit model was selected basing on the lowest AIC, the lowest residual deviance and the significant pairwise test (*P* < 0.001)

**Table 3:** Estimates and 95% confidence intervals from the A-P–C model of LBW, PTB, and SGA

Factor	LBW		PTB		SGA	
**Age**	**Rate**	**95%CI**	**Rate**	**95%CI**	**Rate**	**95%CI**
15	0.068	( 0.063 ~ 0.073)	0.090	( 0.084 ~ 0.095)	0.274	( 0.263 ~ 0.285)
16	0.060	( 0.056 ~ 0.064)	0.080	( 0.076 ~ 0.084)	0.247	( 0.239 ~ 0.255)
17	0.053	( 0.050 ~ 0.056)	0.072	( 0.069 ~ 0.075)	0.223	( 0.217 ~ 0.229)
18	0.047	( 0.045 ~ 0.049)	0.064	( 0.062 ~ 0.066)	0.201	( 0.197 ~ 0.205)
19	0.042	( 0.040 ~ 0.043)	0.057	( 0.055 ~ 0.059)	0.182	( 0.179 ~ 0.185)
20	0.037	( 0.036 ~ 0.038)	0.051	( 0.050 ~ 0.053)	0.164	( 0.161 ~ 0.167)
21	0.033	( 0.032 ~ 0.034)	0.046	( 0.045 ~ 0.047)	0.148	( 0.145 ~ 0.151)
22	0.030	( 0.029 ~ 0.031)	0.042	( 0.041 ~ 0.043)	0.132	( 0.130 ~ 0.134)
23	0.027	( 0.026 ~ 0.028)	0.040	( 0.039 ~ 0.041)	0.117	( 0.115 ~ 0.119)
24	0.026	( 0.025 ~ 0.027)	0.040	( 0.039 ~ 0.041)	0.106	( 0.105 ~ 0.107)
25	0.026	( 0.025 ~ 0.027)	0.041	( 0.040 ~ 0.042)	0.098	( 0.096 ~ 0.099)
26	0.026	( 0.025 ~ 0.027)	0.042	( 0.041 ~ 0.043)	0.091	( 0.089 ~ 0.093)
27	0.026	( 0.025 ~ 0.027)	0.042	( 0.041 ~ 0.044)	0.086	( 0.084 ~ 0.088)
28	0.027	( 0.026 ~ 0.027)	0.043	( 0.042 ~ 0.044)	0.082	( 0.081 ~ 0.084)
29	0.028	( 0.027 ~ 0.028)	0.046	( 0.045 ~ 0.047)	0.080	( 0.079 ~ 0.081)
30	0.030	( 0.029 ~ 0.031)	0.049	( 0.048 ~ 0.050)	0.078	( 0.077 ~ 0.080)
31	0.031	( 0.030 ~ 0.032)	0.052	( 0.050 ~ 0.053)	0.075	( 0.074 ~ 0.077)
32	0.033	( 0.032 ~ 0.034)	0.055	( 0.053 ~ 0.057)	0.072	( 0.071 ~ 0.073)
33	0.035	( 0.034 ~ 0.036)	0.059	( 0.057 ~ 0.061)	0.070	( 0.068 ~ 0.071)
34	0.037	( 0.036 ~ 0.039)	0.063	( 0.062 ~ 0.065)	0.068	( 0.067 ~ 0.069)
35	0.040	( 0.039 ~ 0.042)	0.068	( 0.066 ~ 0.070)	0.067	( 0.066 ~ 0.068)
36	0.043	( 0.042 ~ 0.045)	0.074	( 0.072 ~ 0.076)	0.066	( 0.065 ~ 0.067)
37	0.046	( 0.045 ~ 0.048)	0.079	( 0.077 ~ 0.082)	0.065	( 0.064 ~ 0.067)
38	0.050	( 0.048 ~ 0.052)	0.086	( 0.083 ~ 0.088)	0.065	( 0.063 ~ 0.066)
39	0.054	( 0.052 ~ 0.056)	0.093	( 0.090 ~ 0.096)	0.064	( 0.062 ~ 0.066)
40	0.058	( 0.055 ~ 0.060)	0.100	( 0.097 ~ 0.104)	0.063	( 0.061 ~ 0.065)
41	0.062	( 0.059 ~ 0.065)	0.108	( 0.104 ~ 0.112)	0.063	( 0.060 ~ 0.065)
42	0.067	( 0.063 ~ 0.071)	0.117	( 0.111 ~ 0.122)	0.062	( 0.059 ~ 0.064)
43	0.072	( 0.067 ~ 0.077)	0.126	( 0.120 ~ 0.132)	0.061	( 0.059 ~ 0.064)
44	0.077	( 0.072 ~ 0.083)	0.136	( 0.128 ~ 0.144)	0.060	( 0.058 ~ 0.063)
**Period**	**RR**	**95%CI**	**RR**	**95%CI**	**RR**	**95%CI**
2004	1.035	( 1.027 ~ 1.043)	1.020	( 1.014 ~ 1.026)	1.028	( 1.023 ~ 1.032)
2009	0.970	( 0.960 ~ 0.979)	0.979	( 0.972 ~ 0.987)	0.986	( 0.981 ~ 0.991)
2014	0.987	( 0.979 ~ 0.995)	0.998	( 0.992 ~ 1.005)	0.970	( 0.965 ~ 0.974)
2019	1.065	( 1.048 ~ 1.082)	1.027	( 1.014 ~ 1.040)	1.088	( 1.077 ~ 1.099)
**Cohort**	**RR**	**95%CI**	**RR**	**95%CI**	**RR**	**95%CI**
1960	0.711	( 0.647 ~ 0.780)	0.693	( 0.643 ~ 0.747)	1.818	( 1.716 ~ 1.926)
1961	0.718	( 0.658 ~ 0.783)	0.704	( 0.657 ~ 0.754)	1.753	( 1.661 ~ 1.851)
1962	0.725	( 0.669 ~ 0.786)	0.715	( 0.670 ~ 0.762)	1.691	( 1.608 ~ 1.778)
1963	0.732	( 0.679 ~ 0.789)	0.726	( 0.684 ~ 0.771)	1.631	( 1.557 ~ 1.708)
1964	0.739	( 0.690 ~ 0.792)	0.738	( 0.699 ~ 0.779)	1.573	( 1.507 ~ 1.641)
1965	0.747	( 0.701 ~ 0.795)	0.749	( 0.713 ~ 0.787)	1.517	( 1.458 ~ 1.577)
1966	0.754	( 0.712 ~ 0.798)	0.761	( 0.728 ~ 0.796)	1.463	( 1.411 ~ 1.515)
1967	0.762	( 0.723 ~ 0.802)	0.773	( 0.743 ~ 0.805)	1.410	( 1.366 ~ 1.457)
1968	0.769	( 0.734 ~ 0.806)	0.785	( 0.757 ~ 0.815)	1.360	( 1.322 ~ 1.400)
1969	0.777	( 0.745 ~ 0.810)	0.798	( 0.772 ~ 0.825)	1.312	( 1.278 ~ 1.346)
1970	0.785	( 0.755 ~ 0.815)	0.811	( 0.786 ~ 0.836)	1.265	( 1.236 ~ 1.295)
1971	0.792	( 0.764 ~ 0.821)	0.823	( 0.800 ~ 0.847)	1.220	( 1.195 ~ 1.246)
1972	0.800	( 0.773 ~ 0.828)	0.836	( 0.813 ~ 0.860)	1.177	( 1.154 ~ 1.199)
1973	0.808	( 0.781 ~ 0.836)	0.850	( 0.827 ~ 0.874)	1.135	( 1.114 ~ 1.155)
1974	0.818	( 0.790 ~ 0.846)	0.865	( 0.841 ~ 0.889)	1.095	( 1.076 ~ 1.115)
1975	0.829	( 0.802 ~ 0.858)	0.881	( 0.858 ~ 0.904)	1.061	( 1.042 ~ 1.080)
1976	0.845	( 0.818 ~ 0.872)	0.899	( 0.875 ~ 0.922)	1.035	( 1.017 ~ 1.053)
1977	0.864	( 0.835 ~ 0.894)	0.916	( 0.891 ~ 0.943)	1.021	( 1.004 ~ 1.039)
1978	0.884	( 0.852 ~ 0.917)	0.924	( 0.897 ~ 0.951)	1.020	( 1.001 ~ 1.040)
1979	0.899	( 0.870 ~ 0.929)	0.915	( 0.892 ~ 0.938)	1.022	( 1.003 ~ 1.042)
1980	0.910	( 0.880 ~ 0.940)	0.917	( 0.894 ~ 0.941)	1.018	( 1.004 ~ 1.033)
1981	0.931	( 0.904 ~ 0.959)	0.952	( 0.929 ~ 0.976)	1.013	( 0.997 ~ 1.030)
1982	0.947	( 0.911 ~ 0.985)	0.975	( 0.945 ~ 1.006)	1.012	( 0.991 ~ 1.034)
1983	0.950	( 0.910 ~ 0.991)	0.956	( 0.924 ~ 0.990)	1.007	( 0.986 ~ 1.028)
1984	0.975	( 0.951 ~ 1.001)	0.971	( 0.954 ~ 0.989)	1.001	( 0.986 ~ 1.017)
1985	**Reference**					
1986	0.993	( 0.975 ~ 1.012)	1.003	( 0.979 ~ 1.027)	1.000	( 0.988 ~ 1.011)
1987	0.977	( 0.939 ~ 1.017)	0.996	( 0.966 ~ 1.028)	0.996	( 0.977 ~ 1.017)
1988	0.980	( 0.943 ~ 1.019)	0.990	( 0.964 ~ 1.017)	0.989	( 0.968 ~ 1.011)
1989	0.991	( 0.960 ~ 1.023)	0.987	( 0.962 ~ 1.013)	0.975	( 0.958 ~ 0.991)
1990	0.994	( 0.964 ~ 1.025)	0.987	( 0.962 ~ 1.012)	0.951	( 0.936 ~ 0.966)
1991	0.989	( 0.960 ~ 1.019)	0.988	( 0.963 ~ 1.013)	0.921	( 0.907 ~ 0.935)
1992	0.980	( 0.951 ~ 1.011)	0.990	( 0.965 ~ 1.017)	0.889	( 0.875 ~ 0.902)
1993	0.971	( 0.939 ~ 1.004)	0.993	( 0.965 ~ 1.021)	0.857	( 0.842 ~ 0.871)
1994	0.961	( 0.925 ~ 0.999)	0.996	( 0.964 ~ 1.028)	0.826	( 0.810 ~ 0.842)
1995	0.952	( 0.911 ~ 0.995)	0.998	( 0.963 ~ 1.035)	0.796	( 0.779 ~ 0.815)
1996	0.943	( 0.896 ~ 0.992)	1.001	( 0.960 ~ 1.043)	0.768	( 0.748 ~ 0.789)
1997	0.934	( 0.880 ~ 0.990)	1.003	( 0.958 ~ 1.051)	0.741	( 0.718 ~ 0.763)
1998	0.924	( 0.865 ~ 0.988)	1.006	( 0.955 ~ 1.060)	0.714	( 0.690 ~ 0.739)
1999	0.915	( 0.850 ~ 0.986)	1.008	( 0.952 ~ 1.069)	0.688	( 0.662 ~ 0.716)
2000	0.907	( 0.835 ~ 0.985)	1.011	( 0.948 ~ 1.078)	0.664	( 0.635 ~ 0.694)
2001	0.898	( 0.820 ~ 0.983)	1.014	( 0.945 ~ 1.087)	0.640	( 0.610 ~ 0.672)
2002	0.889	( 0.805 ~ 0.982)	1.016	( 0.942 ~ 1.097)	0.617	( 0.585 ~ 0.651)
2003	0.880	( 0.790 ~ 0.981)	1.019	( 0.938 ~ 1.106)	0.595	( 0.562 ~ 0.630)
2004	0.872	( 0.776 ~ 0.979)	1.021	( 0.935 ~ 1.116)	0.574	( 0.539 ~ 0.611)

*A-P–C* age-period-cohort, *LBW* low birth weight, *PTB* preterm birth, *SGA* small for gestational age

## Discussion

In this retrospective study based on data obtained from the birth registry in Shanghai from 2004 to 2020, we investigated the prevalence of LBW, PTB, and SGA, and observed the secular trends. We examined the independent effects of maternal age, delivery period, and maternal birth cohort on the trends in LBW, PTB, and SGA births, and further explored the modification effect by parity. The “U-shaped” relationship between maternal age and LBW/PTB was examined in this study. Mothers born before the 1980s had a lower incidence of PTB than those born in more recent years. Meanwhile, the risk of SGA declined with advancing age and in cohorts since 1960. However, there were no obvious fluctuant trends in the three birth outcomes by period, suggesting that the observed temporal changes were mostly influenced by the maternal birth cohort.

The estimated prevalence rates of LBW, PTB, and SGA in Shanghai were lower than the national prevalence, and close to that of other developed cities or regions in China. For instance, in urban districts in Beijing, the percentage of LBW fluctuated around 4.0% [22], and the estimated rate of LBW and PTB in the Guangdong province was 4.14% and 4.16%, respectively [23]. The prevalence of SGA was 10.1% in 13 developed cities in China [24]. Our findings also indicate a significantly rising trend of LBW and PTB, and a declining trend of SGA, which is consistent with previous studies [8, 13, 25].

Our findings on the association between maternal age, birth cohort, and LBW/PTB are also consistent with previous studies [26, 27]. Extremes of maternal age increased the incidence of LBW/PTB, suggesting that natural ageing or social environments, or an interaction of both, should account for the association. Generally, older women were believed to have more obstetric complications, which in turn was a high-risk factor for LBW/PTB. Young women < 18 years old were also more likely to have a higher risk of ABOs because of physical immaturity and irregular prenatal care, especially for teenage pregnancies [28, 29].

We noted that the birth cohort (maternal experience and background of growth) would remarkably affect LBW/PTB. With the postponement of the birth year among women born before 1985, the risk of LBW/PTB increased, which may reflect maternal nutrition, environmental exposure, obstetric interventions, and pregnancy complications [30–33]. Although the improving maternal socioeconomic status (SES) and nutritional status decreased the risk of LBW/PTB, the consequent air pollutants, obesity and the stresses and strains of life would contribute to the increasing trend [31, 34, 35]. Previous research has demonstrated the casual and dose–response relationship between active/passive maternal smoking and alcohol consumption during pregnancy, and the risks of LBW/PTB [36–39]. More than an estimated 20% of women of childbearing age (18–39) and 6.5% of pregnant women consumed alcohol in China, and the prevalence of consuming alcohol had increased among both men and women since 2002 [40–42]. A nationwide cross-sectional study estimated that, of the pregnant women in China, 0.56% were smokers and 4.43% were ex-smokers [43]. Studies have shown that maternal exposure to fine particulate matter was associated with LBW/PTB [44]. The increasing use of assisted reproductive technology may be another factor contributing to the rise in LBW/PTB [45]. Also, prenatal complications may increase the risk of LBW/PTB [32, 33]. An observational study based on national registry revealed that the proportion of women with prenatal complications and medical diseases increased from 14.4% to 23.8% 66%and from 3.5% to 11.2%, respectively, from 2012 to 2018 [13].

Interestingly, advanced maternal age was associated with lower rates of SGA in this study, while prior studies have reported conflicting results [18, 46]. The debatable association could be attributed to several reasons: (1) Classifying SGA by various fetal growth curves or birth weight percentiles would lead to differences in the prevalence of SGA [24]. The birth weight percentiles based on healthy populations, could be more effective for recognizing neonates with intrauterine growth restriction or infant mortality [47, 48]. (2) Both domestic and international studies have indicated that maternal age over 40 years was associated with higher risk of SGA, while relatively less advanced maternal age (30–39) was associated with lower risk, compared with maternal age of those in their twenties [49, 50].The small proportion of pregnant women aged over 40 years in our study made it difficult to observe the independent effect of advanced maternal age on SGA. (3) Ethnic differences in the risk factors for SGA across populations [18, 51].

Although it is difficult to explain the decreased trend of SGA, there are some theories to explain this. Firstly, we hypothesised that mothers could have benefited from accessible prenatal interventions and nutritional improvements. Due to the rapid developments in SES, the nutritional and health status of urban residents has greatly improved, and more fertility policies have been promoted [13]. Previous studies in China showed that the decreasing trend of SGA was accompanied by a significant reduction in caesareans and an increasing frequency of antenatal visits over the past decade, meaning that women born in more recent cohorts were unlikely to have SGA births [13]. Secondly, the specific contribution of factors associated with SGA have changed over time [52]. For instance, income inadequacy and being a recent immigrant were risk factors unique to SGA [34, 53], and poor maternal mental health was a risk factor specific to LBW/PTB [54]. Meanwhile, maternal education and parity were associated with both SGA and PTB, and the effect on SGA was greater than PTB [53, 55], which could be the interpretation of the different trend observed in primiparous mothers. Finally, SGA was essentially a different conception from other ABOs. LBW, the traditionally used metric of intrauterine nutrition, overlapped a great deal with PTB, and those infants heavier than 2500 g might also be premature [56]. Hence, identifying newborn babies with intrauterine growth restriction by means of LBW/PTB could be arbitrary [56]. In settings with a high proportion of SGA births but neither LBW nor PTB and the high mortality risk of term-SGA in Asia, SGA would be more sensitive and suitable for identifying intrauterine growth restriction and tracking neonatal health [3, 56, 57]. In summary, SGA and LBW/PTB were distinct but related pregnancy outcomes, and the risk factors related to these outcomes had both differences and similarities, which could account for the different trends of SGA.

It is worth noting that parity might play a role in the association between maternal age and SGA, which has been reported in prior related studies [50, 53]. The slight rising rates in advanced primiparas was consistent with a study conducted in America, which identified that, among primiparous mothers, maternal age ≥ 30 had higher rates of SGA compared with those aged 20–29 years [50]. In contrast to LBW and PTB, we found a reduced incidence rate of SGA in multiparas at the same age, compared to primiparas over 25 years of age, suggesting a different pathogenesis for LBW, PTB and SGA. A retrospective study conducted in China also found that, compared with primiparas aged 25–29, multiparas aged ≥ 35 were at lower risk, examining the combined effects of maternal age and parity on SGA [53]. One potential explanation was that LBW and PTB were more likely to link with placental and oocyte defectivity, whereas SGA might mainly attribute to intrauterine nutritional deficiency [53]. To sum up, our results suggested that parity may affect advanced maternal age and the risk of SGA, which might be due to the lower placenta blood stream and smaller uterine cavity in primiparous mothers [58, 59]. Regarding birth cohort, however, the rising trend beginning from 1960 to 1985 in primiparas is more difficult to explain, and additional studies are needed to explore it.

To the best of our knowledge, several studies have identified maternal APC effect on PTB and SGA [18, 26, 60, 61]. Although our study aimed to analyse the temporal influence of LBW, PTB, and SGA births, several important limitations should be considered. First, due to the lack of other determinants of ABOs, including maternal smoking, gestational weight gain, pregnancy complications, and paternal factors, we were unable to elucidate the mechanisms of maternal age and cohort effects on ABOs [36, 62]. Second, the estimated gestational age, based on the first date of a woman's last menstrual period and not on ultrasound-based methods, may not accurately classify PTB/SGA infants. Although misclassification might influence the results mentioned above, it was unlikely to contribute to the temporal trends entirely. Third, the data was collected from a single birth registry database, which does not represent the nationwide population. However, Shanghai is a megacity with a large population (almost 25 million), which could be representative of the other developed cities in China and other developed Asian countries.

Our study demonstrates independent effects of maternal age, delivery period, and maternal birth cohort on trends in LBW, PTB, and SGA. Within the context of the universal 2-child policy, more women of advanced age prefer to raise a second child in China [63]. Although older women obtained better education and higher SES through social selection, they were more likely to suffer from obstetric complications. Both young and advanced mothers are more likely to have LBW/PTB; accordingly, more prenatal care and public education should be provided to younger and older pregnant women.

## Conclusions

In summary, we found strong maternal age and birth cohort effects on LBW, PTB, and SGA, suggesting that younger and older pregnant women should be key target population groups for perinatal care and treatment. Moreover, there was a continuous increase in the incidence rates of LBW and PTB, encouraging the need to formulate public health intervention and prevention policies in the developed areas of China. Among women in the same age groups, those born in more recent years had a lower risk of SGA. More knowledge of how these trends were associated with LBW, PTB, and SGA in China is required.

## Supplementary Information

**Additional file 1: Supplementary Table 1. **Birthweight percentiles by sex and gestational age. **Supplementary Figure 1. **Flowchart of study population selection. **Supplementary Figure 2. **Age-period-cohort influences on trends in LBW by parity.** Supplementary Figure 3. **Age-period-cohort influences on trends in PTB by parity.** Supplementary Figure 4. **Age-period-cohort influences on trends in SGA by parity.

## Data Availability

The datasets used and analysed in the study are available from the corresponding author upon reasonable request.

## Abbreviations

ABOs: Adverse birth outcomes
LBW: Low birth weight
PTB: Preterm birth
SGA: Small for gestational age
SCDC: Shanghai Municipal Centre for Disease Control and Prevention
APC: Age-period-cohort
CI: Confidence interval
RR: Risk ratio
SDs: Standard deviations
SES: Socioeconomic status
