# Supplementary material for: Secular trends of low birth weight, preterm birth, and small for gestational age in Shanghai from 2004 to 2020: an age-period-cohort analysis
Source: BMC Pregnancy Childbirth. 2023 Jul 26;23:540. doi: 10.1186/s12884-023-05799-9 (PMC10373378; doi:10.1186/s12884-023-05799-9)
Supplement: Supplementary file 1 — Additional file 1: Supplementary Table 1. Birthweight percentiles by sex and gestational age. Supplementary Figure 1. Flowchart of study population selection. Supplementary Figure 2. Age-period-cohort influences on trends in LBW by parity. Supplementary Figure 3. Age-period-cohort influences on trends in PTB by parity. Supplementary Figure 4. Age-period-cohort influences on trends in SGA by parity. [file 12884_2023_5799_MOESM1_ESM.docx]

**Supplementary Table 1** Birthweight percentiles by sex and gestational age

| Gestational age  (weeks) | Male | | |  | Female | | |
| --- | --- | --- | --- | --- | --- | --- | --- |
|  | P_10_ | Mean | P_90_ |  | P_10_ | Mean | P_90_ |
| 24 | 602 | 694 | 787 |  | 583 | 669 | 756 |
| 25 | 702 | 811 | 919 |  | 681 | 782 | 883 |
| 26 | 814 | 940 | 1066 |  | 789 | 906 | 1024 |
| 27 | 938 | 1082 | 1227 |  | 908 | 1043 | 1178 |
| 28 | 1072 | 1237 | 1402 |  | 1038 | 1193 | 1347 |
| 29 | 1216 | 1404 | 1592 |  | 1179 | 1354 | 1529 |
| 30 | 1371 | 1583 | 1794 |  | 1328 | 1526 | 1723 |
| 31 | 1534 | 1771 | 2008 |  | 1487 | 1708 | 1929 |
| 32 | 1705 | 1968 | 2231 |  | 1652 | 1897 | 2143 |
| 33 | 1881 | 2171 | 2462 |  | 1823 | 2094 | 2364 |
| 34 | 2061 | 2379 | 2697 |  | 1997 | 2294 | 2590 |
| 35 | 2242 | 2588 | 2934 |  | 2172 | 2495 | 2818 |
| 36 | 2422 | 2795 | 3169 |  | 2346 | 2695 | 3044 |
| 37 | 2597 | 2998 | 3399 |  | 2517 | 2891 | 3265 |
| 38 | 2766 | 3193 | 3620 |  | 2680 | 3079 | 3477 |
| 39 | 2925 | 3376 | 3828 |  | 2834 | 3255 | 3677 |
| 40 | 3072 | 3545 | 4019 |  | 2976 | 3418 | 3861 |
| 41 | 3202 | 3696 | 4190 |  | 3103 | 3564 | 4025 |
